# Higher-order Brain Areas Associated with Real-time Functional MRI Neurofeedback Training of the Somato-motor Cortex

**DOI:** 10.1016/j.neuroscience.2016.04.034

**Published:** 2018-05-15

**Authors:** Tibor Auer, Wan Ilma Dewiputri, Jens Frahm, Renate Schweizer

**Affiliations:** a Biomedizinische NMR Forschungs GmbH at the Max-Planck-Institute for Biophysical Chemistry, Göttingen, Germany; b MRC Cognition and Brain Sciences Unit, Cambridge, United Kingdom; c Department of Neuroscience, Universiti Sains Malaysia, 16150 Kubang Kerian, Kelantan, Malaysia; d Pusat PERMATApintar Negara, Universiti Kebangsaan Malaysia, 43600 Bangi, Selangor Malaysia

**Keywords:** aMCC, anterior midcingulate cortex, ART, Aligned Rank Transform, FSD, Feedback Signal Difference, GLM, general linear model, NFB, Neurofeedback, PPI, psycho-physiological interaction, rt-fMRI, real-time functional magnetic resonance imaging, SMA, supplementary motor area, SMC, somato-motor cortex, Functional magnetic resonance imaging, functional connectivity, neurofeedback, skill-learning, motor imagery

## Abstract

Neurofeedback (NFB) allows subjects to learn self-regulation of neuronal brain activation based on information about the ongoing activation. The implementation of real-time functional magnetic resonance imaging (rt-fMRI) for NFB training now facilitates the investigation into underlying processes.

Our study involved 16 control and 16 training right-handed subjects, the latter performing an extensive rt-fMRI NFB training using motor imagery. A previous analysis focused on the targeted primary somato-motor cortex (SMC). The present study extends the analysis to the supplementary motor area (SMA), the next higher brain area within the hierarchy of the motor system. We also examined transfer-related functional connectivity using a whole-volume psycho-physiological interaction (PPI) analysis to reveal brain areas associated with learning.

The ROI analysis of the pre- and post-training fMRI data for motor imagery without NFB (transfer) resulted in a significant training-specific increase in the SMA. It could also be shown that the contralateral SMA exhibited a larger increase than the ipsilateral SMA in the training and the transfer runs, and that the right-hand training elicited a larger increase in the transfer runs than the left-hand training. The PPI analysis revealed a training-specific increase in transfer-related functional connectivity between the left SMA and frontal areas as well as the anterior midcingulate cortex (aMCC) for right- and left-hand trainings. Moreover, the transfer success was related with training-specific increase in functional connectivity between the left SMA and the target area SMC.

Our study demonstrates that NFB training increases functional connectivity with non-targeted brain areas. These are associated with the training strategy (i.e., SMA) as well as with learning the NFB skill (i.e., aMCC and frontal areas). This detailed description of both the system to be trained and the areas involved in learning can provide valuable information for further optimization of NFB trainings.

## Highlights

• The increased functional connectivity between the SMA and aMCC indicates a mediator role of the aMCC in NFB learning.
• Successful regulation of the targeted SMC is correlated with its functional connectivity with the non-targeted SMA.
• The involvement of the aMCC and frontal areas and the path of influence support the skill-learning concept of NFB.
• The investigation into the intrinsic property of the system to be trained is helpful to understand how NFB works.

## Introduction

Neurofeedback (NFB) allows subjects to learn self-regulation of neuronal brain activation, which is normally not under volitional control. This can be achieved in a training, in which subjects find mental strategies for regulation based on the information about the ongoing neuronal activation (NFB signal). This activation is usually measured within one or more target regions (Weiskopf et al., 2004) or across the whole brain (LaConte, 2011). Since NFB trainings have been developed to be valuable therapeutic tools, the main scientific interest lies in their development and optimization, as well as in the adaption to new clinical fields. The underlying mechanisms are less frequently investigated, because of methodological limitations as well as the complexity and diversity of the associated learning processes. The methodological limits are due to the relatively weak spatial localization power of the EEG, even with modern multi-channel EEG systems (Baillet et al., 2001), which is still the main technology used to realize NFB in a clinical setting. The reduced spatial resolution limits the training of specific brain areas, as well as the general investigation into brain areas involved in NFB learning. The emergence of real-time functional magnetic resonance imaging (rt-fMRI) (Cox et al., 1995) nowadays allows for imaging of BOLD activation across the entire brain with millimeter spatial resolution and within a couple of seconds. Although the temporal contingency of the feedback is severely hampered by the delayed hemodynamic response, fMRI nevertheless allows the exploration of NFB learning mechanisms by examining the brain areas involved. This can be studied in successful rt-fMRI NFB trainings as well as by investigating which brain areas/processes are involved when a person learns to gain self-regulation of brain activation. The emerging descriptions will not be only of scientific interest but also of practical consideration: by being able to describe the diversity of processes by which NFB learning is acquired, we can better understand between-subject variability in training success and the role of instructions in refining the search space for an optimal strategy (see also (Strehl, 2014)). Ultimately, this can lead to more effective NFB paradigms.

The relatively novel brain network perspective in rt-fMRI NFB developed out of the possibility of fMRI to not only provide feedback from circumscribed brain areas in real time, but also to allow the already mentioned off-line whole-volume analysis of the data. This led to the question, if brain areas other than the targeted, are also influenced by the NFB training (for an overview see Ruiz et al., 2014). Within the motor network, two independent rt-fMRI feedback studies targeting the premotor cortex could not only show changed or increased activation in the premotor cortex, but also increased activation in motor-related areas such as the supplementary motor area (SMA), the basal ganglia and the cerebellum (Marins et al., 2015) as well as significantly altered interactions of the target region with related regions such as the superior parietal lobe (Zhao et al., 2013).

The present rt-fMRI NFB study also targeted a brain area within the motor system: the somato-motor cortex (SMC). The suggested mental strategy was kinesthetic imagery of separate right- and left-hand movements. The specific design of a relatively large number of trained subjects performing 12 separate NFB training sessions (2 runs each) over a period of 4 weeks allowed for more time for the subjects to train self-regulation than comparable studies using the single-session approach (Yoo et al., 2008, Berman et al., 2012, Zhao et al., 2013, Marins et al., 2015). The analysis of the parameters of this rt-fMRI NFB training showed that 67% of the training runs were efficient; i.e. subjects were able to increase the NFB signal in these training runs. 75% of the subjects were shown to be successful in the post-training transfer run; i.e. they were able to increase the SMC-related signal even without the feedback being present (Auer et al., 2015). Additional analysis into the details of this specific NFB training revealed that there was no general difference in the training or transfer outcome between right-hand and left-hand imagery and that the elevated NFB signal was mainly driven by the up-regulation of the SMC contralateral to the imagined hand movements (Auer et al., 2015). Whole-volume general linear model (GLM) analysis of the pre- and post-training transfer runs showed that the group of trained subjects had a significantly higher increase in activation in the contralateral SMC than the control group. This interaction (i.e. training) effect could not be seen in other brain areas outside this targeted SMC region. This was surprising since NFB learning has been associated with multiple learning processes potentially involving different additional brain areas (Birbaumer et al., 2013).

We therefore extended the analysis of this study to other areas and compared functional connectivity within the transfer runs before and after the training to reveal brain areas associated with performing a NFB training and contributing to the learning of the self-regulation.

Since the NFB training targeted the primary motor and somatosensory cortex and since the whole-volume fMRI analysis showed training effect in this SMC area in the trained group, the first approach was to perform a ROI analysis of the non-targeted, next higher brain area within the hierarchy of the motor system, the SMA. The SMA is involved in complex motor behavior as well as in the planning of motor behavior, thereby directly influencing activation of the primary motor cortex. It has therefore been targeted for a clinical NFB study in Parkinson’s disease (Subramanian et al., 2011). The ROI analysis performed on the SMA probed the fMRI activations in the non-targeted contra- and ipsilateral SMA during the right- and left-hand NFB training runs, as well as during the pre- and post-training transfer runs. In a second step a psycho-physiological interaction (PPI) analysis was applied to explore the functional connectivity of the SMC and SMA to other brain areas. The PPI can indicate brain area which exhibits similar ‘physiological’ fMRI time course as the SMC/SMA seed region, but only under the specific ‘psychological’ condition of the NFB transfer (interaction). The third step consisted of a PPI analysis which further included the degree of NFB training success, which represents the pre- to post-training increase in NFB signal amplitude in the transfer runs. Our aim was to test which brain areas are involved in the successful regulation – without receiving feedback – of the targeted SMC after the training.

## Experimental procedures

Seventeen healthy young adults (10 male, mean age: 26 ± 3.3, range: 20–31 years) performed the NFB training. One had to be excluded due to overt hand movement during the training. Sixteen subjects were right handed, one subject showed ambidexterity (laterality index: 20) (overall laterality index: 79 ± 21, based on Edinburgh Inventory (Oldfield, 1971)). The control group consisted of sixteen demographically matched right-handed individuals (seven male, mean age: 27 years ± 3.5, range: 22–34 years, laterality index: 87 ± 12) (see our previous study (Auer et al., 2015) for detailed information). All experimental procedures conformed fully to the institutional guidelines and were approved by the institutional Review Board. Written informed consent was obtained from all subjects before each MRI examination.

Subjects participating in the training group underwent 14 MRI examinations: one pre-training session, 12 training sessions, and one post-training session.

The pre-training session consisted of a whole-brain structural T1-weighted MRI measurement, a fMRI functional localizer measurement involving finger movements of both hands to delineate the target regions-of-interest for NFB within the left and right SMC and two fMRI transfer runs equivalent to the NFB training runs, but without any feedback (“non-feedback” fMRI) to assess the ability of the subjects to control their SMC activities for each hand before the NFB training. An additional single whole-brain EPI scan was acquired in the same slice orientation as the partial-volume EPI fMRI data to improve the registration procedures during data analysis.

The 12 training sessions were spread over 4 weeks of three sessions per week, scheduled at the same time of the day on Mondays, Wednesdays, and Fridays to ensure consistency. In each training session two fMRI runs of right-hand training and two fMRI runs of left-hand training were conducted. The order of right- and left-hand trainings was randomized. No training outside the scanner was performed.

The post-training session consisted of the same measurements as the pre-training session: a whole-brain structural T1-weighted MRI scan, the fMRI functional localizer in which the subjects were instructed to carry out the overt movement task as in the pre-training session and two fMRI training runs without feedback for each hand. The subjects did not receive any additional instruction for the overt movement task (e.g. to pay special attention or to employ their optimized strategy during the task).

The control group only performed the pre-training session and, after 4 weeks, the post-training session. No training was carried out for them.

### MRI

**Fig. 1:**
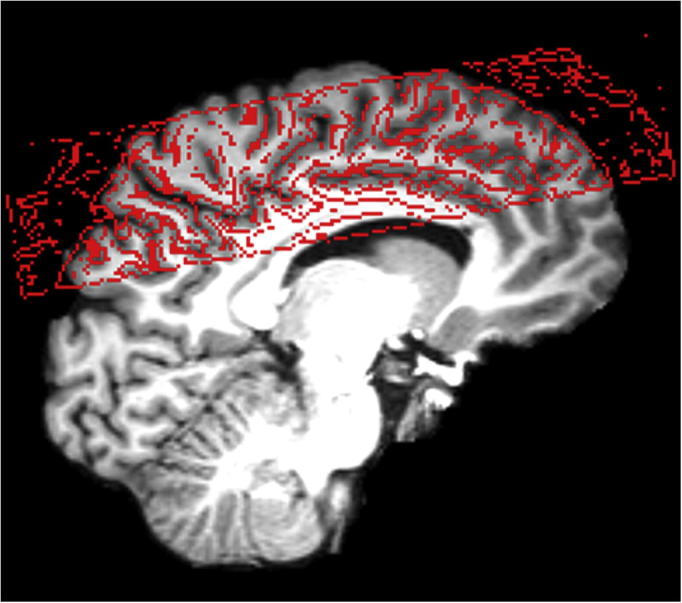
Registration of a functional image (red outlines) to the structural image (background) for a typical subject. The overlay demonstrates the limited field-of-view for fMRI.

MRI was conducted at 3 T (Tim Trio, Siemens Healthcare, Erlangen, Germany) using a 12-channel head coil for signal reception. Structural whole-brain T1-weighted MRI employed a nonselective inversion-recovery 3D FLASH sequence (TR = 2530 ms, TE = 3.65 ms, flip angle 7°, TI = 1100 ms) at a nominal resolution of 1.3 × 1.0 × 1.3 mm^3^. All functional MRI measurements were based on a gradient-echo EPI sequence (TR = 2000 ms, TE = 36 ms, flip angle 70°) with 2-mm isotropic spatial resolution (22 slices, AC-PC orientation) yielding acquired voxel sizes (8 mm^3^) far smaller than in previous fMRI-based NFB studies (20–50 mm^3^) (Zotev et al., 2011, Weiskopf, 2012). Because we did not want to decrease temporal resolution, the price was the limited brain coverage (Fig. 1). Real-time data export (Weiskopf et al., 2005) allowed for access of the data by our in-house NFB toolbox which performed online fMRI analysis (see below). In parallel, all images were also stored in the standard image database and corrected for motion as supplied by the manufacturer (Siemens Healthcare, Erlangen, Germany). These images were used for offline whole-volume analysis. For each subject, a single whole-brain EPI measurement with the same orientation as the fMRI measurements was obtained (TR/TE = 7210/36 ms, flip angle 70°, 2-mm isotropic resolution, 80 slices) to optimize registration of the partial-volume fMRI EPI measurements to the structural whole-brain image. For each subject, the field-of-view (FOV) and slice positions of the pre-training session were stored and reapplied (Siemens AutoAlign Head) in all subsequent sessions to minimize the spatial difference between datasets.

### Functional localizer

**Fig. 2:**
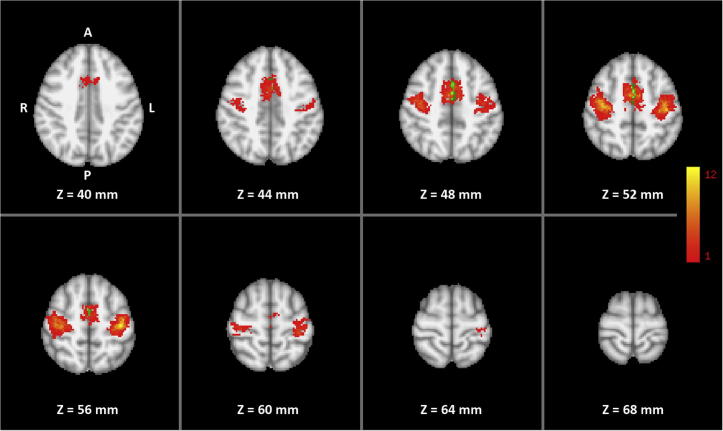
Overlap of individual target regions of the 16 trained subjects (MNI template) with colors indicating the number of subjects (1 to maximum of 12) showing activation during the pre-training overt finger movement task at a particular voxel. Green voxels are included in both right and left hemispheric SMA.

Left and right SMC were identified individually based on fMRI of a bilateral sequential finger opposition task (Strother et al., 1995) comprising eight cycles of active movements (12 s/6 images) and motor rest (18 s/9 images). Subjects were instructed to perform the finger task with both hands with a frequency of 1–2 Hz. Performance was monitored through a video surveillance system. fMRI data were analyzed at a single-subject level using FEAT bundled in FSL 4.1.6 (FMRIB Centre, Department of Clinical Neurology, University of Oxford). Preprocessing consisted of brain extraction, motion correction, and high-pass filtering, but no spatial filtering was applied to preserve the fine-scale spatial resolution. A GLM was then applied to the data with a double gamma hemodynamic response function. A temporal derivative was added to the design to increase robustness to a variable hemodynamic delay. In all cases, thresholding was accomplished by means of the two-threshold (TT) method, which does not require a certain degree of smoothness (Baudewig et al., 2003, Auer and Frahm, 2009) with an upper threshold of *p* = 0.0001 and a lower threshold of *p* = 0.05. For each subject, significant BOLD activation clusters within left and right SMC and SMA were selected in native space based on the individual anatomy (Fig. 2 and Table 1).

**Table 1:** Cluster extent (number of voxels) and coordinates (in mm, MNI space) for local maxima and centers of gravity within right and left somato-motor cortex (SMC)

Region	Cluster extent	Local Maxima			Center of Gravity		
	Mean ± SD	X	Y	Z	X	Y	Z
Left SMC	130 ± 34	−38 ± 5	−19 ± 6	54 ± 3	−37 ± 3	−19 ± 4	54 ± 2
Right SMC	144 ± 32	40 ± 4	−17 ± 6	52 ± 4	39 ± 3	−17 ± 4	52 ± 2
Left SMA	245 ± 99	−3 ± 3	−5 ± 6	54 ± 4	−4 ± 2	−2 ± 6	51 ± 3
Right SMA	268 ± 125	3 ± 4	−3 ± 6	54 ± 4	4 ± 2	0 ± 5	50 ± 3

### fMRI during NFB training

Each session of fMRI NFB training consisted of four separate runs: two involving motor imagery of the right hand and two involving the left hand. A training run started with a baseline period without a task (30 s, 15 images) and a control period (40 s, 20 images), followed by four cycles of training period (30 s, 15 images) and control period (40 s, 20 images) (total: 5:50 min, 175 images). Short visual markers (500 ms) indicated the beginning and end of the training periods. Subjects were instructed to find cognitive strategies to increase their brain activation in the specific brain areas related to finger movement, and examples were given to the subject about previously successful strategies for both training (e.g. imagining well-trained movements) and control phases (e.g. imagining landscapes or covert calculating). Subjects were also instructed to avoid deliberate changes in their general arousal state other than in the given imagery task (deCharms et al., 2004) and to keep their breathing rate as constant as possible. It was strongly emphasized that any change had to be achieved without any overt movement. Because no MRI-compatible EMG was available, lack of overt movement during the imagined movement task was verified by video surveillance (Lee et al., 2009).

During both the training and control periods, subjects obtained visual feedback via LCD goggles (VisuaStim XGA, Resonance Technology Inc., Northridge, CA, USA) in real time. The feedback was presented by means of a horizontal blue rectangular bar (feedback-meter) on a white screen. The base of the bar was centered in the middle of the screen and the bar could change length toward the right or left side. For the right-hand training, the subjects’ task was to find a motor imagery strategy to increase the length of the bar to the right side; while for the left-hand training, to increase the length of the bar to the left side of the screen. During the control periods the feedback meter had to be kept as low as possible. Subjects were also aware that there was a latency of 8–10 s in the feedback (about 4–6 s due to the BOLD response and 4 s due to image acquisition and reconstruction, data transfer, preprocessing (motion correction), and analysis).

During post-scanning interviews, possible improvements on the strategy were also discussed (e.g. imagining finger oppositions with random order and/or higher speed), but no training outside the scanner was asked for.

Real-time analysis and presentation of the feedback was accomplished using an in-house toolbox for NFB implemented in MATLAB (MathWorks, Natick, MA, USA). Each scan was automatically registered to the first scan of the functional localizer acquired in the pre-training session. Continuous motion correction was realized with real-time registration based on the SPM5 Realign function (Welcome Trust Centre for Neuroimaging, University College London). For each of the two ROIs (right and left SMC) obtained from the individual functional localizer scan, a normalized signal (NS) is calculated for each time point with reference to the mean of the last 10 time points of the previous control period according to

(1) $${\text{NS}}_{t}=(S_{t}/S_{\text{previous}\_\text{control}}-1)\times 100$$

where *S_t_* and *S*_previous_control_ correspond to the signal intensity at time point *t* and during the previous control period, respectively.

To increase the robustness and ensure insensitivity of the normalized signal to signal fluctuations around zero, a double logistic-like function with values ranging from 21 for −2 NS to 2 NS and a flat center between −0.25 and 0.25 NS was applied (Fig. 2 in (Dewiputri and Auer, 2013)).

Similar to a study by Lee and co-workers (Lee et al., 2009) the feedback signal given to the subjects was the Feedback Signal Difference (FSD) between the normalized signal from two different ROIs, the left and right SMC (laterality training):

(2) $${\text{FSD}}_{t}=\text{NS}\_{\text{Left}}_{t}-\text{NS}\_{\text{Right}}_{t}$$

This resulted in a positive FSD for successful right-hand training (elongation of the bar in the visual feedback to the right side) and a negative FSD for successful left-hand training (elongation of the bar to the left side).

To obtain parameters describing changes in BOLD activation of SMA during the training, fMRI data of the training sessions were also analyzed offline using MATLAB. GLM was performed on the time courses extracted from the individual ROIs (left and right SMA), and parameter estimates (% signal change) for the contralateral SMA (% signal change_contra_) and the ipsilateral SMA (% signal change_ipsi_) were computed.

To ensure normal distribution of the investigated parameters, Aligned Rank Transform (ART) (Salter and Fawcett, 1993) as implemented in ARTool (Wobbrock et al., 2011) was applied to % signal changes. Rank Transform (Conover and Iman, 1981) is commonly used to allow for parametric ANOVA on non-normally distributed values after transformed to ranks. However, it is accurate only for testing main effects (Salter and Fawcett, 1993). By aligning values to tested effects (main or interaction) before Rank Transform, ART results in ranks which can also be used to test any interactions (Mansouri, 1998).

ART-ed % signal change in contra- and ipsilateral SMAs was compared with a 3-way within-subject ANOVA with the factors HAND (left vs right), HEMISPHERE (contra- vs ipsilateral), and TIME (24 training runs).

The fMRI data of the pre- and post-training transfer runs were analyzed identically to the training data. ART-ed % signal change in the contra- and ipsilateral SMAs and the non-feedback transfer runs were obtained for the training and the control group. They were compared with a 4-way mixed ANOVA applying the within-subject factors HAND (left vs right), HEMISPHERE (contra- vs ipsilateral), and TIME (pre- vs. post-training), as well as the between-subject factor GROUP (training vs. control).

### PPI

A voxel-wise whole-volume fMRI analysis of the PPI (Friston et al., 1997, Kim and Horwitz, 2008) was performed for the transfer runs using FEAT. Preprocessing steps consisted of those employed for the functional localizer (see above). Modest spatial filtering with a FWHM = 5 mm was applied to allow for better registration (Maisog and Chmielowska, 1998) and to suppress the influence of within- and between-subject variability (Mikl et al., 2008). Because the partial-volume functional datasets covered only a part of the brain, a three-stage linear registration using FLIRT (Jenkinson and Smith, 2001) was performed to register the partial-volume EPI images via the single whole-brain EPI image and the anatomical T1-weighted 3D image into standard MNI space. For each subjects’ pre- and post-training transfer runs, time courses were extracted from the peaks of maximal activation in the ipsi- and contralateral SMC and SMA. The single-subject model consisted of the present experimental task (psychological component), the time courses referring to the activity level in the seed regions (physiological component), and the interaction between the task and seed activity (PPI). This PPI is usually referred to as ‘task-related functional connectivity’. Since the task was brain regulation in our transfer run, we can consider PPI as the ‘transfer-related functional connectivity’. Transfer-related functional connectivity was estimated for right- and left-hand trainings separately employing the ipsi- and contralateral SMC and SMA as seed ROIs (eight analyses). The interactions were further examined at the group level using a 2-way mixed ANOVA (GROUP and TIME) with mixed effects model FLAME1 + 2 (Woolrich et al., 2004). Z (Gaussianized T) and statistic images were thresholded using clusters determined by Z > 2 and a cluster significance threshold of *p* = 0.05 corrected for multiple comparisons.

We further extended the second-level model with the degree of NFB training success defined by the pre- to post-training difference in the NFB signal, which was contra- vs ipsilateral SMC % signal change. This model tested where pre- to post-training change in transfer-related functional connectivity correlates with the training success (within-group contrast) and whether this correlation is stronger in the training group than in the control group (interaction contrast). Group model was estimated using FLAME1 + 2. Z (Gaussianized T) and statistic images were thresholded using clusters determined by Z > 2 and a cluster significance threshold of *p* = 0.05 corrected for multiple comparisons.

Anatomical labeling of every result was performed with reference to the Harvard–Oxford cortical and subcortical structural atlases (http://fsl.fmrib.ox.ac.uk/fsl/fslwiki/Atlases). Laterality index LI was calculated according to the equation (Eq. (3)):

(3) $$\text{LI}=(n{\text{Voxel}}_{L}-n{\text{Voxe}}_{R})/(n{\text{Voxel}}_{L}+n{\text{Voxe}}_{R})$$

## Results

### Non-target SMA activation

#### SMA activation in pre- and post-training transfer runs

**Fig. 3:**
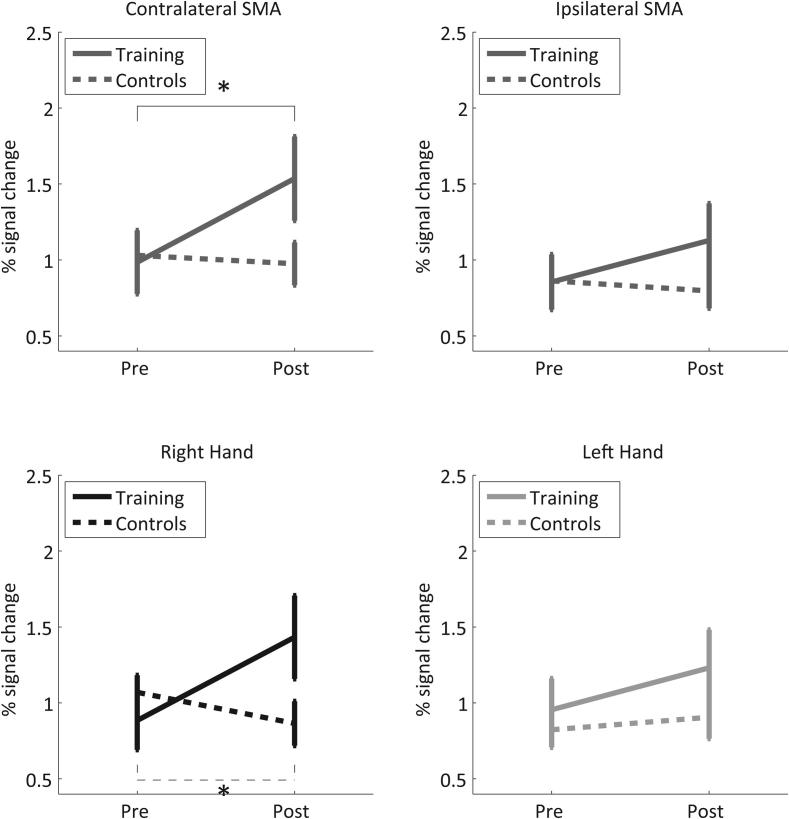
Training effect in SMA. Visualization of the effect of the hemisphere (top) and the trained hand (bottom) on the TIME × GROUP interaction. ^∗^Significant (*p* < 0.05).

To describe the potential effect of the NFB training of SMC on the non-target area SMA, a 4-way mixed ANOVA was conducted on the ART-ed SMA % signal change with the factors HEMISPHERE (ipsilateral, contralateral), TIME (pre-training, post-training), GROUP (trained, control) and HAND (right, left). The main effect HEMISPHERE (*F*(1,30) = 35.65, *p* < 0.001) shows that the contra- and the ipsilateral SMAs are not affected equally, the contralateral SMA showing generally larger activation. The additional interaction HEMISPHERE × TIME (*F*(1,30) = 8.85, *p* = 0.006) indicates that the training influences are higher on the contralateral SMA, where the pre- to post-training increase in BOLD activation is larger than in the ipsilateral SMA. Finally, the interaction HEMISPHERE × TIME × GROUP (*F*(1,30) = 4.81, *p* = 0.036) (Fig. 3 top) reveals that the HEMISPHERE × TIME interaction was larger in the training group. Although no significant main effect HAND was present, there was a significant interaction HAND × TIME × GROUP (*F*(1,30) = 6.12, *p* = 0.019) (Fig. 3 bottom) indicating that the pre- to post-training difference of the BOLD % signal change of the SMA (independent of the hemisphere) was larger for the training group and this TIME × GROUP interaction was larger for the right hand.

### SMA activation during training

To describe the activation in the ipsilateral and contralateral SMA in the trained subjects across the 24 training runs, a 3-way ANOVA with the factors HEMISPHERE (ipsilateral, contralateral), HAND (right, left) and TIME (1–24 training runs) was performed on the ART-ed SMA % signal change.

**Fig. 4:**
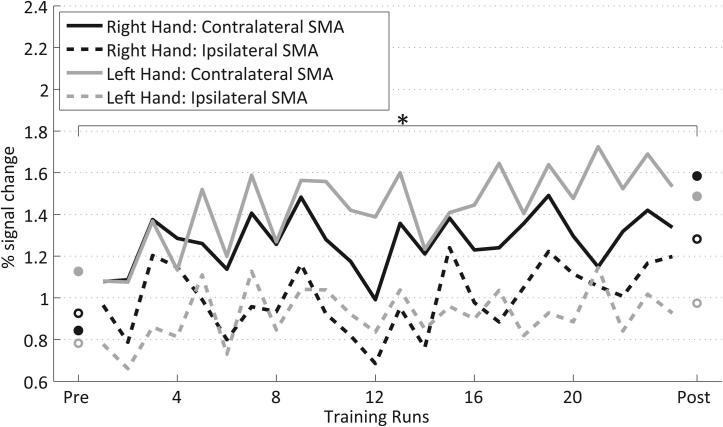
Percent signal change in contralateral (solid) and ipsilateral (dashed) SMA of trained subjects across NFB runs for the right (black) and left (gray) hand. ^∗^Significant (*p* < 0.01) pre- to post-training change in contralateral SMA activity for the right-hand training.

Similar to the analysis comparing the transfer results, a significant main effect HEMISPHERE (*F*(1,15) = 49.45, *p* < 0.001) was detected, showing a larger BOLD activation in the contralateral SMA. Again similar to the transfer, the significant interaction HEMISPHERE × TIME (*F*(24,360 Huynh-Feldt corrected) = 1.88, *p* = 0.028) (Fig. 4) documents a larger increase in BOLD activation in the contralateral SMAs compared to the ipsilateral SMAs over the time course of the training. The additional significant interaction HAND × TIME (*F*(24,360 Huynh-Feldt corrected) = 1.74, *p* = 0.049) reveals a different time course of SMA BOLD activation during the training of the two hands.

### PPI-based connectivity

Eight PPI analyses tested which brain areas in the acquired MRI volume showed pre- to post-training increase in transfer-related functional connectivity with either of the 4 seed areas SMCc, SMCi, SMAc, and SMAi. The right- and left-hand training data were analyzed separately (hence eight analyses in total). The 2-way ANOVAs comprised the factors TIME (pre-training, post-training) and GROUP (trained, control).

**Fig. 5:**
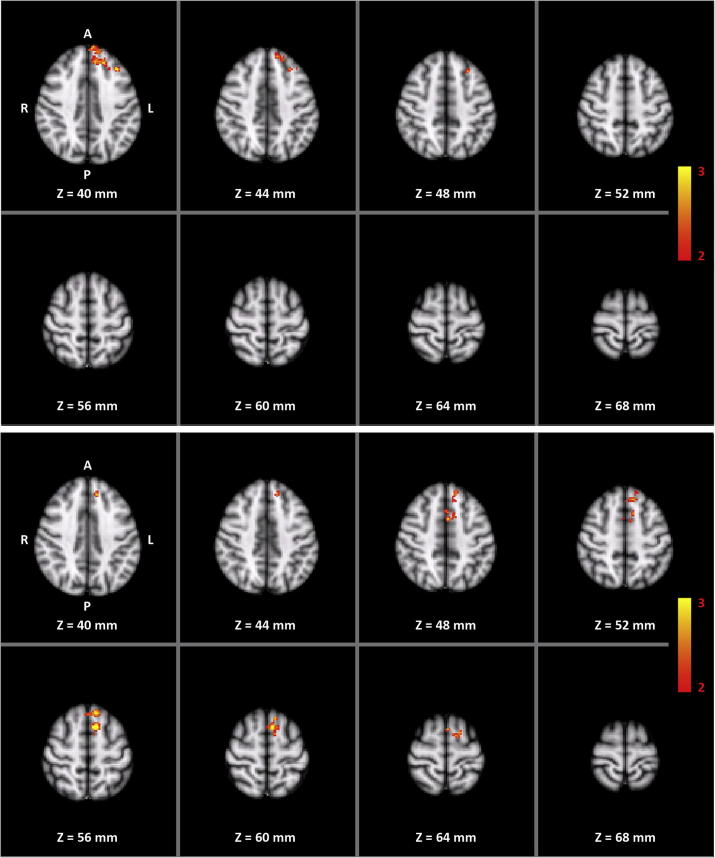
Two-way mixed ANOVA of the whole-volume PPI for the right-hand (top) and left-hand (bottom) motor imagery without feedback. Left SMA was used as seed region for analyzing both the right- and left-hand data. Color indicates pre- to post-training increase in transfer-related functional connectivity with the left SMA significantly higher for the training group than for the control group (interaction TIME × GROUP).

No brain area showed a training-specific change in pre- to post-training increase in transfer-related functional connectivity with the targeted SMCs. However, frontal and prefrontal areas, as well as the anterior midcingulate cortex (aMCC) showed a pre- to post-training increase in transfer-related functional connectivity with the left SMA (results not shown) for both right- and left-hand trainings. This increase was larger in the training group than in the control group only for the frontal areas and the aMCC (Fig. 5 and Table 2). Regardless of which hand the subjects mentally trained, brain areas showing increased PPI were strongly lateralized to the left hemisphere (overall laterality of the significant clusters: right-hand training LI = 0.8; left-hand training LI = 0.9).

**Table 2:** Areas (number of voxels and coordinates in MNI space) showing increased transfer-related functional connectivity (i.e. PPI) with left SMA

Training	Region	Number of voxels	Local Maxima			
			Z-Max	X	Y	Z
Right	Frontal Pole L	242	2.83	−12	38	38
	Frontal Pole R	33	3.05	6	58	20
	Superior Frontal Gyrus L	154	3.55	−8	44	32
	Superior Frontal Gyrus R	13	2.55	4	52	32
	Middle Frontal Gyrus L	91	3.07	−36	28	40
	Paracingulate Gyrus (aMCC) L	37	3.19	−8	36	38
	Paracingulate Gyrus (aMCC) R	12	2.59	2	40	36
Left	Frontal Pole L	21	2.59	−12	38	42
	Superior Frontal Gyrus L	302	3.66	−6	12	58
	Superior Frontal Gyrus R	11	2.43	2	28	54
	Juxtapositional Lobule Cortex (SMA) L	63	2.9	−2	6	48
	Juxtapositional Lobule Cortex (SMA) R	3	2.09	2	6	52
	Paracingulate Gyrus (aMCC) L	37	2.52	−2	14	48
	Paracingulate Gyrus (aMCC) R	4	2.29	2	16	50

**Fig. 6:**
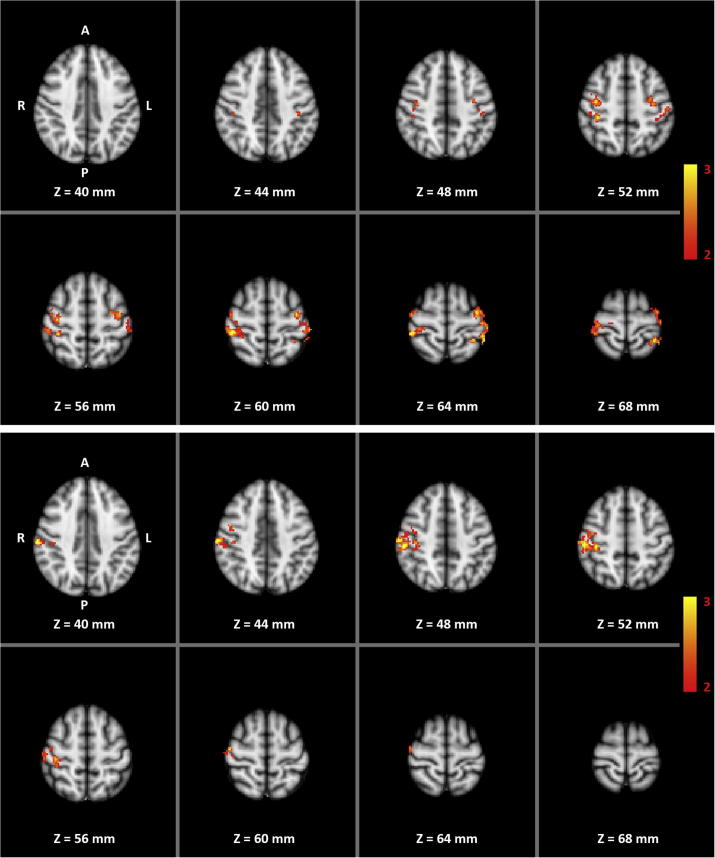
Whole-volume correlation analysis between increase in PPI and training success for right-hand motor imagery without feedback. Left SMA was used as seed region for analyzing both the right- and left-hand data. Color indicates significant correlation within the training group (top) or correlation significantly stronger within the training group than within the control group (bottom).

Additional PPI analysis tested where pre- to post-training changes in functional connectivity correlated with transfer success. For the right-hand training, the pre- to post-training increase in transfer-related functional connectivity between the left SMA and several areas in the central and postcentral gyri bilaterally correlated with the transfer success in the training group (Fig. 6 top and Table 3). Between the left SMA and the right (ipsilateral) SMC, this correlation was stronger in the training group than in the control group (Fig. 6 bottom and Table 3).

**Table 3:** Areas (number of voxels and coordinates in MNI space) showing correlation between training success and transfer-related functional connectivity (i.e. PPI) with left SMA for the right-hand training

Contrast	Region	Number of voxels	Local Maxima			
			Z-Max	X	Y	Z
Training	Precentral Gyrus L	259	3.5	64	59	69
	Precentral Gyrus R	260	3.2	27	56	63
	Postcentral Gyrus L	241	3.2	59	42	73
	Postcentral Gyrus R	437	3.6	23	46	68
	Superior Parietal Lobule L	121	3.5	60	40	72
	Superior Parietal Lobule R	47	3.2	34	41	74

Training vs Control	Precentral Gyrus R	94	2.9	22	56	66
	Postcentral Gyrus R	374	3.9	19	50	61
	Supramarginal Gyrus R	161	3.5	14	51	56

## Discussion

The exploration of non-targeted but training-related brain areas was started within the motor system focussing on the SMA, and then extended into the overall brain space covered by the fMRI volume. The ROI analysis of the SMA BOLD activation during the 24 training runs as well as during the pre- and post-training transfer runs indicated significant training-specific increase in the SMA in addition to SMC reported in a previous study (Auer et al., 2015). In contrast to SMC, SMA showed a hemispheric and a hand training difference. The contralateral SMA exhibited a larger increase than the ipsilateral SMA in the training as well as in the transfer runs and the right-hand training elicited a larger increase in the transfer run than the left-hand training. The whole-volume, pre- and post-training transfer PPI analysis searching for brain areas with similar activation patterns as the SMC and SMA revealed no brain activation related to the SMC; but the left-hemispheric aMCC and frontal regions showed activation related to the left-hemispheric SMA. An additional whole-volume PPI analysis, taking into account the gain in the ability to regulate the targeted SMC in the transfer run, indicated that this transfer success was related with the training-specific increase in transfer-related functional connectivity between the left SMA and the target area SMC.

The increase in SMA activation indicates its involvement in the training, but the signal increase from pre- to post-training transfer was smaller than in the targeted SMC, which explains why it could not be seen in the general pre- to post-training whole-brain GLM analysis (Auer et al., 2015). The involvement of the non-targeted SMA is not unexpected, being in the hierarchy of the motor system involved in motor planning of executed movements as well as motor imagery. Executed movements and motor imagery not only activates similar cortical regions, but do share neural networks involving the contralateral motor cortex (BA4), dorsal premotor cortex (PMd), parietal areas and SMA, with the SMA being part of a network which is equally involved in motor imagery as well as in executed motor movement (Sharma and Baron, 2013). A functional connectivity study shows bi-directional functional connectivity between the SMA and SMC for motor execution as well as motor imagery (Gao et al., 2011). The training effect in the non-targeted SMA can therefore be explained either as a bottom-up effect from the target SMC or as a top-down effect of the SMA on the SMC. A comparison of the trained subjects’ average group time courses of the SMA and SMC activations (Auer et al., 2015) across the 24 training trials showed that the SMA showed higher activation than the SMC right from the start. This initial SMA activation can be attributed to the instructed motor imagery, which at the beginning was not strongly associated with SMC activation. Toward the end of the training both areas showed an increase in activation, although this increase was less in the SMA than in the targeted SMC. In the transfer condition successful transfer was associated with an increase in functional connectivity between SMA and SMC, suggesting a possible increase in the hierarchical top-down influence of SMA on SMC as a consequence of a successful NFB training.

Another finding in the SMA analysis was that the right- and the left-hand training had different effects on SMA activation, possibly reflecting the handedness asymmetry seen in overt movement and motor imagery. SMA activity during left-hand training had a different time-course across the training and a slight tendency to be higher than in the right-hand training. This changed in the pre- to post-transfer comparison, where the right-hand training resulted in higher post-training SMA activity than left-hand training. These SMA results are in general contrasting the SMC results, where respective differences could neither be seen in training efficiency nor in transfer success, indicating that the ability to regulate the SMC as a result of the NFB trainings was not different for the right- and the left-hand training. From the present analysis it remains unclear how the influence of hand asymmetry on SMA results relates to the asymmetry-independent SMC outcome of the right- and left-hand NFB trainings.

The PPI analysis revealed which region(s) showed an increase in functional connectivity with the assigned seed region(s) in a NFB-specific way. Here an increase in functional connectivity between the left SMA and aMCC as well as frontal areas was demonstrated. The aMCC is a natural candidate to play a prominent role in NFB training. It is responsible for – among other processes – salience (Seeley et al., 2007), reward evaluation (Rushworth and Behrens, 2008), cognitive control (Shackman et al., 2011) and updating a predictive model to reflect changes in the environment (O’Reilly et al., 2013). The aMCC, involved in cognitive and emotional processing, has been in the focus since the beginning of fMRI-NFB (Weiskopf et al., 2003). More recent fMRI NFB studies of cognitive networks also reported increased functional connectivity between target areas and the aMCC during the NFB sessions (Ruiz et al., 2011, Zotev et al., 2011). Volumetric measures of the aMCC were also shown to predict the general responsiveness to frontal-midline theta EEG-NFB training (Enriquez-Geppert et al., 2013). Moreover, Ros and co-workers found up-regulation of the dorsal anterior cingulate cortex and midcingulate cortex at around 30 min after an EEG-NFB training of the Pz alpha rhythm, when subjects mostly employed focused visual attention as strategy (Ros et al., 2013). It is important to emphasize that all of the abovementioned studies trained the aMCC directly or cognitive processes which could also activate the aMCC. It is therefore not possible to decide whether the aMCC was involved in learning to regulate the targeted area or simply as a part of the trained network/cognitive process. Consequently, these findings do not imply that the aMCC plays a more general role in NFB. Our study, not targeting a cognitive brain area but the SMC, shows the involvement of the aMCC despite the fact that this “cognitive” brain area is not part of the motor network and has no direct link to the SMC (Hanakawa et al., 2008). The pre- to post-training transfer increase in functional connectivity between the aMCC and the SMA in the trained subjects confirms that the potential involvement of the aMCC influences the SMC via the SMA and not directly; which also supports the top-down hypothesis in NFB training. Interestingly, the aMCC areas showed a strong lateralization to the left hemisphere (Table 2). Although the results indicate that there may be a link between the lateralization of the SMA and the aMCC as well as other frontal areas, our data provide no further evidence whether it is linked to the handedness-related hemispheric asymmetry within the motor system, or because the lateralization of one brain area determines the lateralization of the others.

While our study does not allow inference on the role of the basal ganglia due to limited brain coverage, it provides evidence for the role of the aMCC in the NFB training of the SMC. It indicates increased cognitive influence over the trained process, which supports the explicit skill-learning concept of NFB and that this skill-learning is mediated by the aMCC. The increased functional connectivity in the post-training transfer between the aMCC and the left SMA but not with either SMC implies that the increased cognitive influence was enforced not directly on the target area but at a higher level area eliciting a top-down control over the target area. Interestingly, the effect lateralizes to the left hemisphere (Table 2) and it is detectable only in the left SMA being in the contralateral hemisphere of the dominant hand. Moreover, the strength in connectivity between the left SMA and the target area has been also increased regardless of whether it is situated in the ipsi- or contralateral hemisphere to the left SMA.

## Conclusion

Although NFB attracts growing interest from neuroscientists and clinicians alike, there are only few studies investigating the mechanism of its effect on brain functions. Some evidence points to the importance of the cortical–basal ganglia loops ([Bibr b0085][Bibr b0085], Birbaumer et al., 2013). Our study directs the attention to the aMCC as an additional candidate for a “NFB-mediator area” in the skill-learning concept of NFB. Our results also indicate that the intrinsic property of the system to be trained is essential to understand how NFB works. This, furthermore, can help us to find an explanation for the between-subject variability of the training success and, finally, may allow for the optimization of NFB paradigms.

## References

[b0005] Auer T., Frahm J. (2009). Functional MRI using one- and two-threshold approaches in SPM5. NeuroImage.

[b0010] Auer T., Schweizer R., Frahm J. (2015). Training efficiency and transfer success in an extended real-time functional MRI neurofeedback training of the somatomotor cortex of healthy subjects. Front Hum Neurosci.

[b0015] Baillet S., Mosher J.C., Leahy R.M. (2001). Electromagnetic brain mapping. Signal Processing Magazine. IEEE.

[b0020] Baudewig J., Dechent P., Merboldt K.D., Frahm J. (2003). Thresholding in correlation analyses of magnetic resonance functional neuroimaging. Magn Reson Imaging.

[b0025] Berman B.D., Horovitz S.G., Venkataraman G., Hallett M. (2012). Self-modulation of primary motor cortex activity with motor and motor imagery tasks using real-time fMRI-based neurofeedback. NeuroImage.

[b0030] Birbaumer N., Ruiz S., Sitaram R. (2013). Learned regulation of brain metabolism. Trends Cogn Sci.

[b0035] Conover W.J., Iman R.L. (1981). Rank transformations as a bridge between parametric and nonparametric statistics. Am Stat.

[b0040] Cox R.W., Jesmanowicz A., Hyde J.S. (1995). Real-time functional magnetic resonance imaging. Magn Reson Med.

[b0045] deCharms R.C., Christoff K., Glover G.H., Pauly J.M., Whitfield S., Gabrieli J.D.E. (2004). Learned regulation of spatially localized brain activation using real-time fMRI. NeuroImage.

[b0050] Dewiputri W.I., Auer T. (2013). Functional magnetic resonance imaging (fMRI) neurofeedback: implementations and applications. Malays J Med Sci.

[b0055] Enriquez-Geppert S., Huster R.J., Scharfenort R., Mokom Z.N., Vosskuhl J., Figge C., Zimmermann J., Herrmann C.S. (2013). The morphology of midcingulate cortex predicts frontal-midline theta neurofeedback success. Front Hum Neurosci.

[b0060] Friston K.J., Buechel C., Fink G.R., Morris J., Rolls E., Dolan R.J. (1997). Psychophysiological and modulatory interactions in neuroimaging. NeuroImage.

[b0065] Gao Q., Duan X., Chen H. (2011). Evaluation of effective connectivity of motor areas during motor imagery and execution using conditional Granger causality. NeuroImage.

[b0070] Hanakawa T., Dimyan M.A., Hallett M. (2008). Motor planning, imagery, and execution in the distributed motor network: a time-course study with functional MRI. Cereb Cortex.

[b0075] Jenkinson M., Smith S. (2001). A global optimisation method for robust affine registration of brain images. Med Image Anal.

[b0080] Kim J., Horwitz B. (2008). Investigating the neural basis for fMRI-based functional connectivity in a blocked design: application to interregional correlations and psycho-physiological interactions. Magn Reson Imaging.

[b0085] Koralek A.C., Jin X., Long J.D., Costa R.M., Carmena J.M. (2012). Corticostriatal plasticity is necessary for learning intentional neuroprosthetic skills. Nature.

[b0090] LaConte S.M. (2011). Decoding fMRI brain states in real-time. NeuroImage.

[b0095] Lee J.-H., Ryu J., Jolesz F.A., Cho Z.-H., Yoo S.-S. (2009). Brain-machine interface via real-time fMRI: preliminary study on thought-controlled robotic arm. Neurosci Lett.

[b0100] Maisog J.M., Chmielowska J. (1998). An efficient method for correcting the edge artifact due to smoothing. Hum Brain Mapp.

[b0105] Mansouri H. (1998). Multifactor analysis of variance based on the aligned rank transform technique. Comput Stat Data Anal.

[b0110] Marins T.F., Rodrigues E.C., Engel A., Hoefle S., Basilio R., Lent R., Moll J., Tovar-Moll F. (2015). Enhancing motor network activity using real-time functional MRI neurofeedback of left premotor cortex. Front Behav Neurosci.

[b0115] Mikl M., Mareček R., Hluštík P., Pavlicová M., Drastich A., Chlebus P., Brázdil M., Krupa P. (2008). Effects of spatial smoothing on fMRI group inferences. Magn Reson Imaging.

[b0120] O’Reilly J.X., Schuffelgen U., Cuell S.F., Behrens T.E., Mars R.B., Rushworth M.F. (2013). Dissociable effects of surprise and model update in parietal and anterior cingulate cortex. Proc Natl Acad Sci U S A.

[b0125] Oldfield R.C. (1971). The assessment and analysis of handedness: the Edinburgh inventory. Neuropsychologia.

[b0130] Ros T., Theberge J., Frewen P.A., Kluetsch R., Densmore M., Calhoun V.D., Lanius R.A. (2013). Mind over chatter: plastic up-regulation of the fMRI salience network directly after EEG neurofeedback. NeuroImage.

[b9000] Ruiz S., Buyukturkoglu K., Rana M., Birbaumer N., Sitaram R. (2014). Real-time fMRI brain computer interfaces: self-regulation of single brain regions to networks. Biol Psychol.

[b0135] Ruiz S., Lee S., Soekadar S.R., Caria A., Veit R., Kircher T., Birbaumer N., Sitaram R. (2011). Acquired self-control of insula cortex modulates emotion recognition and brain network connectivity in schizophrenia. Hum Brain Mapp.

[b0140] Rushworth M.F., Behrens T.E. (2008). Choice, uncertainty and value in prefrontal and cingulate cortex. Nat Neurosci.

[b0145] Salter K.C., Fawcett R.F. (1993). The art test of interaction – a robust and powerful rank test of interaction in factorial models. Commun Stat Simulat.

[b0150] Seeley W.W., Menon V., Schatzberg A.F., Keller J., Glover G.H., Kenna H., Reiss A.L., Greicius M.D. (2007). Dissociable intrinsic connectivity networks for salience processing and executive control. J Neurosci.

[b0155] Shackman A.J., Salomons T.V., Slagter H.A., Fox A.S., Winter J.J., Davidson R.J. (2011). The integration of negative affect, pain and cognitive control in the cingulate cortex. Nat Rev Neurosci.

[b0160] Sharma N., Baron J.C. (2013). Does motor imagery share neural networks with executed movement: a multivariate fMRI analysis. Front Hum Neurosci.

[b0165] Strehl U. (2014). What learning theories can teach us in designing neurofeedback treatments. Front Hum Neurosci.

[b0170] Strother S.C., Anderson J.R., Schaper K.A., Sidtis J.J., Liow J.S., Woods R.P., Rottenberg D.A. (1995). Principal component analysis and the scaled subprofile model compared to intersubject averaging and statistical parametric mapping: I. “Functional connectivity” of the human motor system studied with [15O]water PET. J Cereb Blood Flow Metab.

[b0175] Subramanian L., Hindle J.V., Johnston S., Roberts M.V., Husain M., Goebel R., Linden D. (2011). Real-time functional magnetic resonance imaging neurofeedback for treatment of Parkinson’s disease. J Neurosci.

[b0180] Weiskopf N. (2012). Real-time fMRI and its application to neurofeedback. NeuroImage.

[b0185] Weiskopf N., Klose U., Birbaumer N., Mathiak K. (2005). Single-shot compensation of image distortions and BOLD contrast optimization using multi-echo EPI for real-time fMRI. NeuroImage.

[b0190] Weiskopf N., Scharnowski F., Veit R., Goebel R., Birbaumer N., Mathiak K. (2004). Self-regulation of local brain activity using real-time functional magnetic resonance imaging (fMRI). J Physiol Paris.

[b0195] Weiskopf N., Veit R., Erb M., Mathiak K., Grodd W., Goebel R., Birbaumer N. (2003). Physiological self-regulation of regional brain activity using real-time functional magnetic resonance imaging (fMRI): methodology and exemplary data. NeuroImage.

[b0200] Wobbrock J.O., Findlater L., Gergle D., Higgins J.J., Conference A.C.M.C.H.I. (2011). The Aligned Rank Transform for nonparametric factorial analyses using only ANOVA procedures. On human factors in computing systems.

[b0205] Woolrich M.W., Behrens T.E., Beckmann C.F., Jenkinson M., Smith S.M. (2004). Multilevel linear modelling for FMRI group analysis using Bayesian inference. NeuroImage.

[b0210] Yoo S.-S., Lee J.-H., O’Leary H., Panych L.P., Jolesz F.A. (2008). Neurofeedback fMRI-mediated learning and consolidation of regional brain activation during motor imagery. Int J Imag Syst Technol.

[b0215] Zhao X., Zhang H., Song S., Ye Q., Guo J., Yao L. (2013). Causal interaction following the alteration of target region activation during motor imagery training using real-time fMRI. Front Hum Neurosci.

[b0220] Zotev V., Krueger F., Phillips R., Alvarez R.P., Simmons W.K., Bellgowan P., Drevets W.C., Bodurka J. (2011). Self-regulation of amygdala activation using real-time FMRI neurofeedback. PLoS One.

